# Encapsulation of copper phenanthroline within horse spleen apoferritin: characterisation, cytotoxic activity and ability to retain temozolomide†

**DOI:** 10.1039/d3ra07430g

**Published:** 2024-04-29

**Authors:** Maria Letizia Cassioli, Michael Fay, Lyudmila Turyanska, Tracey D. Bradshaw, Neil R. Thomas, Anca Pordea

**Affiliations:** a Faculty of Engineering, University of Nottingham NG7 2RD UK anca.pordea@nottingham.ac.uk; b Nanoscale and Microscale Research Centre, University of Nottingham NG7 2RD UK; c Biodiscovery Institute, School of Pharmacy, University of Nottingham NG7 2RD UK; d Biodiscovery Institute, School of Chemistry, University of Nottingham NG7 2RD UK

## Abstract

Protein capsules are promising drug delivery vehicles for cancer research therapies. Apoferritin (AFt) is a self-assembling 12 nm diameter hollow nanocage with many desirable features for drug delivery, however, control of drug retention inside the protein cage remains challenging. Here we report the encapsulation of copper(ii)-1,10-phenanthroline (Cu(phen)) within the horse spleen AFt (HSAFt) nanocage, by diffusion of the metal through the pores between the protein subunits. Transmission electron microscopy revealed the formation of organised copper adducts inside HSAFt, without affecting protein integrity. These structures proved stable during storage (>4 months at −20 °C). Exposure to physiologically relevant conditions (37 °C) showed some selectivity in cargo release after 24 h at pH 5.5, relevant to the internalisation of AFt within the endosome (60% release), compared to pH 7.4, relevant to the bloodstream (40% release). Co-encapsulation of temozolomide, a prodrug used to treat glioblastoma multiforme, and Cu(phen) enabled entrapment of an average of 339 TMZ molecules per cage. *In vitro* results from MTT and clonogenic assays identified cytotoxic activity of the Cu(phen), HSAFt–Cu(phen) and HSAFt–Cu(phen)–TMZ adducts against colorectal cancer cells (HCT-116) and glioblastoma cells (U373V, U373M). However, the presence of the metal also contributed to more potent activity toward healthy MRC5 fibroblasts, a result that requires further investigation to assess the clinical viability of this system.

## Abstract 2

Copper complexes and temozolomide can be co-encapsulated within apoferritin.

## Graphical abstract

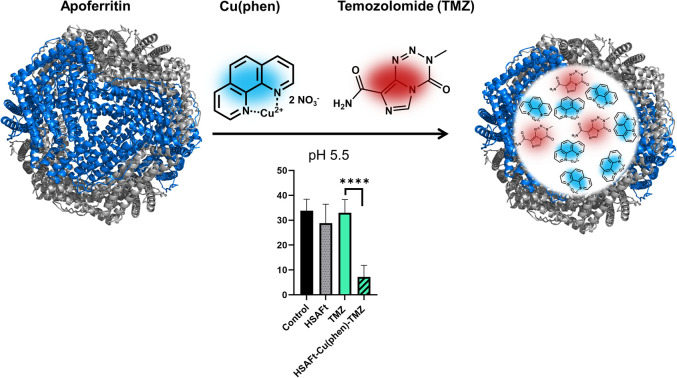

## Introduction

Ferritin (Ft) is an iron storage protein, which forms a self-assembling cage composed of 24 heavy subunits. In its iron-free apoferritin form, this protein cage has been extensively studied as a drug delivery vehicle, due to its size (12 nm external diameter and 8 nm inner cavity) and its biocompatibility. The apoferritin (AFt) shell has been successfully utilised for loading anticancer drug molecules,^1^ catalysts^2^ and nanoparticles.^3^ The most commonly used method for the encapsulation of molecules into AFt follows the disassembly-reassembly route, whereby the cage is dissociated at extreme pH (2.5 or 11), however this approach leads to partial protein denaturation.^4^

Passive diffusion through the two sets of protein channels (0.4 nm wide, 8 pores at the 3 fold symmetry axis; 6 at the 4-fold axis with different transport properties) connecting the surface with the protein core can also be used for loading small drug molecules, *via* the so-called ‘nanoreactor’ route.^5–7^ However, the retention of neutral molecules smaller than maltose inside the capsule remains challenging and there is uncertainty as to whether the drugs are trapped within the protein cavity or accumulating within the protein shell.^4,8,9,10^

Given its iron storage function, the internal surface of AFt is lined with acidic amino acid residues, which can be exploited for the retention of metal complexes (*e.g.* of Pt, Au, Ag, Ru).^11,12^ X-ray crystallography studies confirmed the ability of the AFt subunits to specifically bind to metallodrugs such as cisplatin, through residues lining the protein internal surface and its channels.^5,12,13^ Recently, improved drug retention was achieved by encapsulation of organic drugs pre-complexed with transition metals, by exploiting AFt's ability to sequester metal ions.^4^ For example, complexation of the poly(adenosine diphosphate-ribose) polymerase (PARP) inhibitor olaparib with Cu(ii) and subsequent encapsulation performed by the nanoreactor route yielded seven times more drug loaded inside AFt than the encapsulation of the non-complexed organic molecule.^14^ This approach was also successful in increasing the stability and the loading efficiency of other anticancer agents such as doxorubicin (DOX)^15^ and temozolomide (TMZ)^16^ inside horse spleen AFt (HSAFt). Comparison of co-encapsulation using the disassembly-reassembly route with passive diffusion of DOX into pre-formed maghemite–AFt suggested that the latter (nanoreactor) route mostly led to the drug bound at the surface rather than within the cavity, resulting in higher release.^8^ Similar conclusions were reached when DOX was loaded by diffusion into AFt containing graphene quantum dots and iron as imaging probes, although some internalisation of DOX could not be ruled out.^8^

Copper complexes with a wide range of ligands have been studied for the replacement of Pt from anticancer metallodrugs.^17^ They have been shown to induce production of reactive oxygen species and to damage DNA. For example, Cu(ii) complexes bearing one or more 1,10-phenanthroline ligand(s) or derivatives thereof have been shown to have potent pharmacological activity against several cancers, with some compounds (Casiopeìnas®) currently under clinical evaluation.^18–20^ The planarity of the N–N moiety has been suggested to increase the potency, whilst a variety of inorganic or organic auxiliary ligands was used in an effort to alter biochemical properties or the ability to target DNA. Furthermore, complexes of phenanthroline with other metals, for example gold(iii), have also shown anticancer properties; whilst, their encapsulation into AFt resulted in increased cytotoxicity in cancer cells, compared to non-tumourigenic cells.^21^ To our knowledge, Cu-phenanthroline metallodrugs have not been encapsulated within AFt.

In this work, we investigate the encapsulation of a Cu(ii)-phenanthroline complex (Cu(phen)(NO_3_)_2_) within HSAFt, as a potential vector for the delivery of anticancer agents. The channels formed by the protein cage subunits combined with the protein's natural affinity for metals favour the loading of the compound *via* the nanoreactor route, avoiding the issues related to protein denaturation associated with the reassembly method. We immobilised Cu(ii) within the protein as a metal complex with 1,10-phenanthroline, to form HSAFt–Cu(phen) dense particles that can be stored for months at low temperature (−20 °C). We subsequently characterised HSAFt–Cu(phen) by drug release studies and cytotoxicity assays against colorectal cancer cells and glioblastoma multiforme cells. We also investigate whether the presence of Cu(phen) inside HSAFt can offer additional benefits for the encapsulation and antitumour activity of anticancer drugs such as TMZ.

## Results and discussion

### Preparation and characterisation of Cu(ii)phenanthroline loaded into horse spleen apoferritin (HSAFt)

**Fig. 1:**
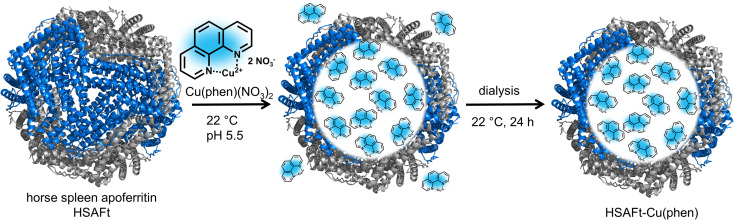
Encapsulation of copper phenanthroline within HSAFt by the nanoreactor route. The removal of the iron core from ferritin yielded the empty HSAFt, which was loaded with the copper complex. The encapsulation by diffusion was performed by incubating the protein with an excess of the complex for 3 h at room temperature and pH 5.5. The sample was then dialysed to ensure the complete removal of residual unencapsulated compound.

We selected the Cu(phen)(NO_3_)_2_ complex for this study, because we hypothesised that its relatively small and simple structure would allow straightforward encapsulation into HSAFt *via* the nanoreactor route (Fig. 1). The complex was synthesised and characterised as previously described by Pessoa and co-workers.^22^ The encapsulation was performed by incubation of the protein and the metal complex under conditions that have previously been reported for the diffusion of organic molecules into AFt, at room temperature and for 3 h in 0.1 M sodium acetate buffer at pH 5.5, where the cage is slightly relaxed and pores are wider.^23,24^ After the encapsulation, excess Cu(phen)(NO_3_)_2_ was removed by dialysis in fresh sodium acetate buffer (see Experimental section).

Inductively coupled plasma mass spectrometry (ICP-MS) analysis of the purified HSAFt–Cu(phen) complex showed an average number of Cu(ii) ions per cage of 225 ± 48 (average of 9 experiments), which was in line with the ratio of 334 copper atoms per HSAFt cage previously reported for the encapsulation of other metal complexes.^16,25,26^ No effect on the integrity of the HSAFt cage structure was observed when the HSAFt–Cu(phen) samples were run on native PAGE (ESI, Fig. SI-1†). The purified sample was stored in 0.1 M sodium acetate buffer at pH 5.5 at −20 °C to allow for other characterisation experiments.

**Fig. 2:**
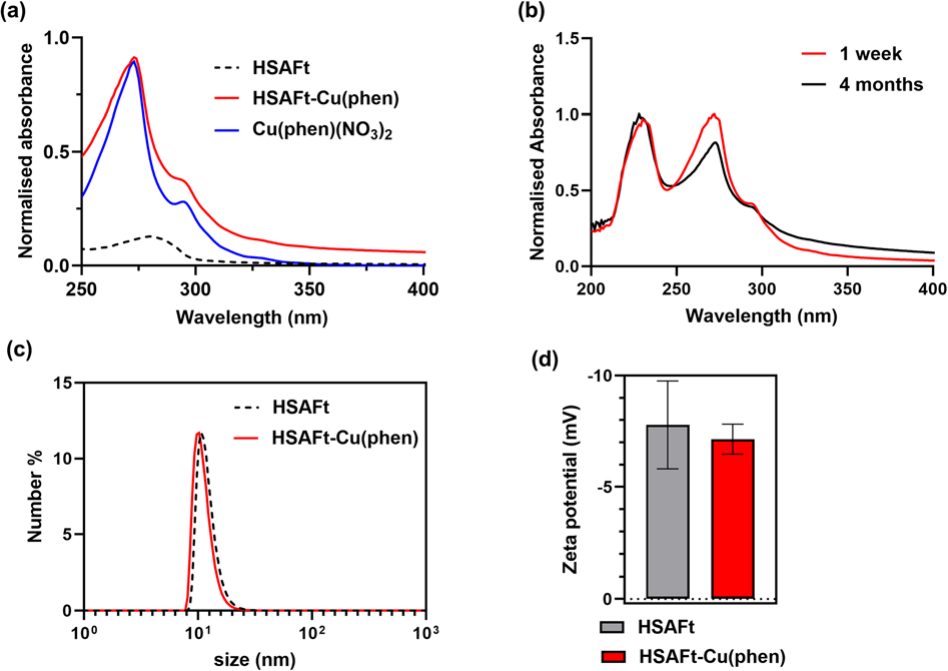
Characterisation of HSAFt–Cu(phen) samples in solution. (a) UV-visible spectra of HSAFt (0.5 mg mL^−1^ in 0.1 M sodium acetate pH 5.5, dotted black line), Cu(phen)(NO_3_)_2_ (0.1 mM in methanol, blue line) and HSAFt–Cu(phen) (0.3 mg mL^−1^ in 0.1 M sodium acetate pH 5.5, red line); (b) UV-visible spectra of HSAFt–Cu(phen) after 1 week (red line) and after 4 months (black line) of storage at −20 °C; (c) hydrodynamic size distribution of HSAFt and HSAFt–Cu(phen) measured by dynamic light scattering and (d) zeta potential values of HSAFt and HSAFt–Cu(phen). Values are reported as mean ± SD (*n* = 3 measurements). No significant difference was observed between control (HSAFt) and sample (HSAFt–Cu(phen)) (*p* > 0.05).

We employ complementary analysis, UV-visible spectroscopy, ICP-MS and TEM imaging, to characterise the encapsulated agents and to confirm the loading of the agents into the AFt capsules. UV-visible spectroscopic analysis performed within 1 week of encapsulation showed that the absorbance spectrum of HSAFt–Cu(phen) was substantially different to that of HSAFt. Similar absorption peaks were observed by comparing the spectrum of the HSAFt–Cu(phen) in buffer with that of Cu(phen)(NO_3_)_2_ in methanol, with two maxima at 230 and 268 nm, together with a shoulder peak at 295 nm (Fig. 2a). The UV-visible absorption spectrum of HSAFt–Cu(phen) after 4 months of storage at −20 °C was similar to the initial spectrum, thus confirming the integrity of the sample under these storage conditions (Fig. 2b). Combined, the UV-visible and the ICP-MS data supported the evidence that both Cu^2+^ and 1,10-phenanthroline were retained within the protein cage after the encapsulation, however at this stage it was difficult to ascertain whether the [Cu(phen)]^2+^ complex remained intact, due to the similarity between the absorption spectra of free and Cu(ii)-bound 1,10-phenanthroline. ICP-MS data obtained from the encapsulation of Cu(NO_3_)_2_ into HSAFt showed that the maximum metal retention was 84 metal ions per protein cage, which was lower than the average measured for HSAFt–Cu(phen). This result suggested that the complexation of Cu to phenanthroline facilitated its loading into HSAFt and that at least some of the Cu^2+^ ions remained bound to the organic ligand upon encapsulation.

Previous work by Merlino and co-workers with gold bipyridine and phenanthroline complexes encapsulated within AFt indicated complex dissociation and binding of Au ions to cysteine and histidine residues, which may also be happening with Cu(phen) complexes.^21^ The same group reported the formation of a small amount of gold nanoparticles under their disassembly – reassembly encapsulation conditions, however in our case no absorption above 500 nm was observed, indicating that copper nanoparticles do not form during our encapsulation experiments.^27^ Other studies have also shown that Cu-phenanthroline complexes undergo speciation in the presence of Cu-binding proteins, such as bovine serum albumin, with a mixture of protein–Cu(phen), protein–Cu and protein–phen species formed at low micromolar Cu concentrations in solution and we hypothesise that a similar situation occurs in the presence of HSAFt.^22^

The DLS and zeta potential analyses of the HSAFt–Cu(phen) sample confirmed it as a monodisperse solution of particles with the hydrodynamic diameter 15.4 ± 2.3 nm and zeta potential of −7.15 ± 0.67 mV (Fig. 2c and d). Comparable values were recorded for HSAFt and HSAFt–Cu(phen) samples, demonstrating that the AFt cage in the HSAFt–Cu(phen) preparation remained intact, with no indication of charged compounds associated to the external surface of the protein. The increase of the hydrodynamic diameter of the sample at pH 5.5 compared to previously published results at pH 7 may come from the cage being relaxed at lower pH, with its channels being larger than at neutral pH conditions.^24^

**Fig. 3:**
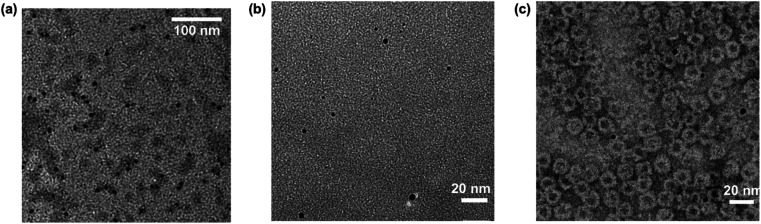
TEM analysis of samples obtained by encapsulation. (a) HSAFt–Cu(phen); (b) HSAFt–Cu(phen)–TMZ; (c) HSAFt–Cu(phen)–TMZ deposited on negatively stained (uranyl acetate) graphene oxide grids.

Transmission electron microscopy (TEM) further confirmed the presence of intact HSAFt cages (Fig. 3), as was reported previously for different cargos.^28,29^ The TEM images taken after one week revealed the presence of electron-dense copper structures that showed a crystalline organisation (Fig. SI-4†). The structures were also observed after 4 months of storage and had a diameter of 8.4 ± 3.4 nm (Fig. 3a), however they could not be visualised 6 months after encapsulation (Fig. SI-2†). Similar structures were not observed in control samples, where Cu(NO_3_)_2_ and 1,10-phenanthroline were encapsulated separately within HSAFt under the same conditions as described above (Fig. SI-3†), thus confirming the hypothesis that under the conditions used, copper accumulation inside HSAFt only occurred upon encapsulation of the Cu(phen) complex.

The effect of the presence of Cu(phen) within HSAFt on the co-encapsulation of the anticancer agent TMZ was assessed next. In a previous publication, members of our team have already reported on the encapsulation of TMZ within HSAFt *via* the nanoreactor route, resulting in improved intracellular drug delivery and *in vitro* cytotoxicity.^23^ Here, TMZ was loaded within HSAFt–Cu(phen) at a ratio of 339 (±13) TMZ molecules per HSAFt cage, somewhat lower when compared to previously reported HSAFt–TMZ formulations (520 TMZ molecules per protein cage) due to the co-encapsulation of both TMZ and Cu(phen). The ICP-MS results indicated a decrease in the copper loading from 339 to 126 ions per HSAFt cage upon TMZ encapsulation, which suggested a loss of copper to favour TMZ accumulation.

TEM analysis confirmed the presence of electron dense copper structures within the HSAFt–Cu(phen)–TMZ samples (Fig. 3b). Furthermore, to detect the protein shell in the TEM images and assess its integrity following the encapsulation, negative staining with uranyl acetate was used. The stained images allowed the visualisation of the AFt shell with an average diameter of 12 ± 2 nm and confirmed its integrity even in the presence of the copper adducts (Fig. 3c). Whilst some copper structures were visualised inside the HSAFt cages, others seemed to be located on the outside of the cages. Presence of copper-bound drug molecules on the exterior of the HSAFt has been reported before.^9^ However, since our DLS results did not reveal any changes of the surface charges following encapsulation process, it is likely that some of the Cu(phen)-containing HSAFt cages were not visible in the negatively stained TEM images.

The images of the HSAFt–Cu(phen) samples after 4 months of storage at −20 °C allowed for analysis of the lattice fringe visible upon magnification on single copper particles (Fig. SI-4†). This arrangement was substantially different from the amorphous iron cores that normally accumulate in Ft and suggested a high crystallinity of the copper adducts, with *d*-spacing for the lattice fringes estimated at 0.25 nm. This lattice spacing is similar to that of CuO nanoparticles (0.264 nm), in which copper and oxygen atoms are packed into a simple cubic unit cell arrangement.^30^ Electron energy loss spectroscopy (EELS) analysis provided additional evidence of copper–oxygen interactions occurring within the protein cage. The EELS spectrum of the HSAFt–Cu(phen) sample showed the energy edges in the 900–950 eV range, which corresponded to those present in reference samples of CuO (Fig. SI-5†).^31^ However, a mechanism for the formation of copper oxide (CuO or Cu_2_O) nanoparticles is difficult to envisage and no change in the colour or in the UV-visible spectrum of the solution was observed to indicate the formation of nanoparticles. Based on the TEM image analysis and the EELS, we hypothesised that the void spaces in the copper crystalline pattern were occupied by oxygen atoms from neighbouring amino acid side chains, such as aspartate and glutamate residues lining the internal surface of the HSAFt cage.^5^ At the same time a central bulk formed by Cu-phenanthroline complexes (*e.g.* [Cu(phen)_*n*_]^2+^ with *n* = 1–3) could form a copper core.^7,32–34^

### Effect of pH and temperature on the formation of HSAFt–Cu(phen)

The influence of buffer pH and temperature on the encapsulation of metal complexes within HSAFt was evaluated next. Variation of buffer composition and pH has previously been shown to improve the encapsulation of large drug molecules such as DOX, without using the disassembly-reassembly strategy.^35^ Similarly, some metal complexes have shown better AFt incorporation when incubated at higher temperatures, such as 45 °C.^6^ A second encapsulation experiment was performed at room temperature using 50 mM glycine at pH 7.4 as the encapsulation buffer. Copper loading was quantified by ICP-MS and revealed the presence of 33 Cu(ii) ions per protein cage, which was lower compared to the samples produced by encapsulation at pH 5.5 (average 225 Cu(ii) ions per cage). This result was further supported by the absence of the Cu(phen) peak at 268 nm in the UV-visible absorption spectrum (Fig. SI-6†). At neutral pH, the HSAFt channels are narrower than at pH 5.5 thus being less permeable to Cu(phen), which could explain the lower encapsulation ratios.^24^

The HSAFt–Cu(phen) sample prepared at pH 7.4 was stored at −20 °C and TEM analysis was carried out at different time intervals. Although a change in the sample over time could be observed, with some electron-dense structures accumulating, the images could not provide clear evidence supporting the formation of copper adducts like those observed in the samples at pH 5.5 (Fig. SI-8†). Furthermore, the low contrast between the circular structures observed and the background made it impossible to magnify the image to a lower nm range to confirm crystalline copper organisation. When the buffer was exchanged to pH 6, TEM analysis showed the formation of copper clusters of 6.3 ± 2.3 nm, confirming that a more acidic pH favoured the copper accumulation. No visible structures were observed when the buffer was exchanged to pH 2, where HSAFt was expected to be disassembled. Overnight incubation at 37 and 50 °C revealed no difference to samples that were not exposed to higher temperature, by TEM imaging (Fig. SI-9†).

### Cargo release studies

**Fig. 4:**
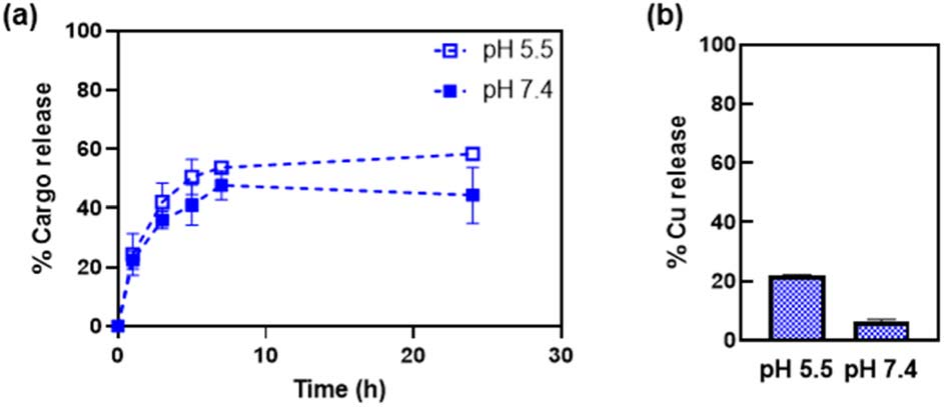
Release of Cu(phen) from HSAFt–Cu(phen), measured by (a) UV-visible spectroscopy and (b) ICP-MS. Samples were exposed to 37 °C at pH 7.4 and pH 5.5. Analyses were performed on the dialysis buffers, to estimate the release of the copper complex (UV-vis) and of copper (ICP-MS) separately. ICP-MS analysis was performed after 24 h incubation. Values are reported as mean ± SD (*n* = 3). Significant differences between pH 5.5 and pH 7.4 (*p* < 0.05) were obtained for values at 7 h and at 24 h.

The release of the Cu(phen) cargo from the HSAFt–Cu(phen) adducts was investigated by exposing the samples to temperature and pH values relevant to physiological conditions, using HSAFt–Cu(phen) formed at pH 5.5. A sample (aged 1–4 months upon storage at −20 °C) was incubated for 24 h at 37 °C and under two different pH conditions (7.4 and 5.5) and the Cu(phen) complex released in the dialysis solution was quantified over time from the absorbance at 268 nm. The acidic pH mimicked the endosomal environment upon internalisation into cancer cells, and the physiological pH was useful to identify any drug leakage in the bloodstream before reaching the tumour site. Up to ∼54% of Cu(phen) was released in solution from HSAFt–Cu(phen) exposed to pH 5.5 within the first 7 h of incubation, whilst only ∼43% was released at pH 7.4 (Fig. 4a). Increasing the exposure time to 24 h led to 60% release at pH 5.5 *vs.* 40% release at pH 7.4. This release profile was consistent with previous release studies and could be explained by the flexibility of the HSAFt cage, whose structure is more ‘relaxed’ at acidic pH leading to the progressive swelling of the subunits and the consequent widening of the channels which accelerates the leakage of its cargo.^1,23,24,36^

The ICP-MS analysis of the dialysis solution after 24 h of incubation detected the largest release of copper ions in the sample exposed to pH 5.5, equivalent to 20% of the total amount of metal encapsulated into HSAFt–Cu(phen), compared to 6% for the sample exposed to pH 7.4 (Fig. 4b). The discrepancy between the UV-vis spectroscopy and the ICP-MS data suggests that the phenanthroline: Cu molar ratio in the released cargo was > 1. This could be explained either by the formation and release of Cu(phen)_*n*_^2+^ complexes (with *n* = 2–3), or by the release of free phenanthroline and retention of Cu^2+^ within the cage. UV-visible spectroscopy could not differentiate between the spectra of these different species. Nonetheless, these data suggest a greater stability of the HSAFt–Cu(phen) at pH 7.4 than at pH 5.5. This pH-dependent ability of AFt to release its cargo within an acidic environment becomes especially relevant upon protein internalisation by cells. Specifically, binding of the heavy (H) subunits of AFt to the transferrin receptor 1 (TfR1), which is overexpressed in cancer cells is followed by internalisation of the cargo-containing protein cage into a vesicle. Upon internalisation the vesicle is converted into an endosome which progressively acidifies to form early endosome (pH 6.5), late endosome (pH 5.5) and then lysosome (pH 4.5). The gradual acidification of the endosome allows for the release of the AFt content as a consequence of the swelling of the protein channels, thus becoming a crucial step to ensure intracellular delivery of encapsulated therapeutic agents.^37^ Thus, our results with Cu(phen) confirm the possibility of a marginally selective release of the protein cage content only after its sequestration by cancer cells and internalisation within the endosome, where the pH is closer to 5.5.

### Antitumour activity of HSAFt–Cu(phen)

**Fig. 5:**
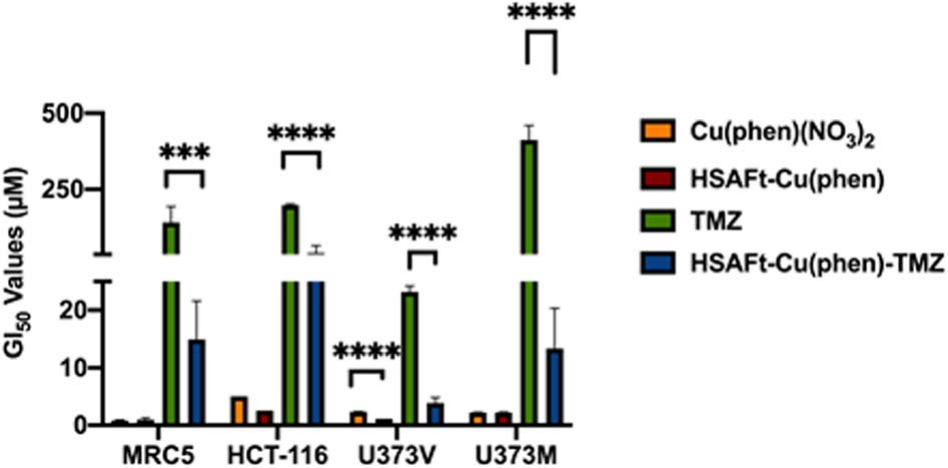
GI_50_ values (μM) of free and encapsulated Cu(phen) for cancer and healthy cells. GI_50_ is expressed as mean ± SEM from three independent trials, where *n* = 8 per trial (internal replicates). Concentrations of test compounds were based on Cu concentration for Cu(phen) and HSAFt–Cu(phen), and on TMZ concentration for HSAFt–Cu(phen)–TMZ.

The MTT cell viability assay was used to determine the *in vitro* activity of Cu(phen)(NO_3_)_2_ and of HSAFt–Cu(phen) on non-tumourigenic human lung fibroblasts (MRC5) and colorectal cancer cells (HCT-116). Cells were exposed to different concentrations of Cu(phen)(NO_3_)_2_, HSAFt or HSAFt–Cu(phen) for 72 h. As previously reported, HSAFt did not show any effect on any of the cell lines analysed (Fig. SI-11†),^23^ and the TMZ alone, used an positive control, had the GI_50_ value comparable to those expected from the literature. On the other hand, both Cu(phen)(NO_3_)_2_ and HSAFt–Cu(phen) displayed a high ability to inhibit cell growth of both cancer and non-cancer cell lines, with GI_50_ values in the low micromolar concentrations (Fig. 5 and SI-11†) and comparable with the reported active concentrations found for other Cu(ii)-phenanthroline complexes against HeLa tumour cells^38^ or colorectal cancer cells.^39^ This showed that encapsulation of Cu(phen) into HSAFt had a limited effect on the activity profile of the metal complex.

**Fig. 6:**
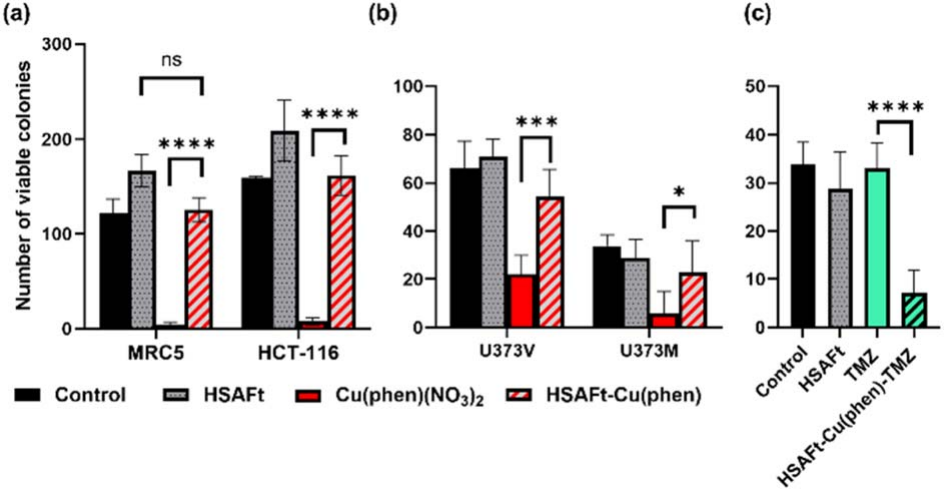
Effect of free and encapsulated Cu(phen) on cancer and healthy cells. (a) MRC5 and HCT-116 cells were seeded at a density of 2 × 10^4^ cells per well, allowed to grow for 24 h and exposed to 0.5 mM test agent for 72 h before counting using a haemocytometer; (b) U373V and U373M cells were seeded in 6-well plates at a low density (400 cells per well) incubated at 37 °C for 24 h before treatment with test agents for 6 days (HSAFt (0.05 μM), Cu(phen)(NO_3_)_2_ (2.5 μM) or HSAFt–Cu(phen) (2.5 μM Cu)), after which the cells were then washed and incubated in fresh media until colonies were observed in the control wells; (c) U373M cells were seeded at 400 cells per well, incubated at 37 °C for 24 h before treatment with test agents for 6 days (HSAFt (0.05 μM), TMZ (20 μM) or HSAFt–Cu(phen)–TMZ (20 μM)). For (b) and (c) colony staining was performed at time of test agent addition (*T*_0_) and following treatment of cells with test agents. Representative cell survival data are shown. Data points are mean ± SD, taken from 3 independent trials where *n* = 2 per trial. Control represents cells incubated with media only until colony staining.

A cell count assay was performed to further assess the effect of the test compounds. In this assay, cells were initially allowed to grow for 24 h, then exposed to 0.5 μM of test compound for 72 h, prior to counting using a haemocytometer. Interestingly, encapsulation within the protein conferred some protection against toxicity, with a stark difference observed between Cu(phen)(NO_3_)_2_ and HSAFt–Cu(phen) (Fig. 6a). This effect was observed with both healthy and cancer cell lines, suggesting that the concentration of HSAFt–Cu(phen) required to kill colorectal cancer is likely to be higher than the minimum safe concentration to avoid toxicity to healthy cells as well.

To further expand on the bioactivity effects of Cu(phen) encapsulated within HSAFt, the free and the encapsulated Cu complex were tested with two lines of isogenic glioblastoma multiforme (GBM) cells, U373V and U373M. GBM is an aggressive brain tumour with poor prognosis and the standard of care for its treatment is the chemotherapy prodrug TMZ. Approximately 50% GBM cells express the enzyme O^6^–methylguanine–DNA methyltransferase MGMT, which confers resistance to treatment with TMZ. The vector control U373V cells used in this study are MGMT-low while the MGMT-transfected U373M cells express high levels of MGMT. The MTT cell viability assays with the GBM cells showed similar GI_50_ values compared to the MRC5 and HCT-116 cells shown above (Fig. 5 and SI-11†). Clonogenic assays were also performed with GBM cells, where they were exposed to 2.5 μM of test compound for 6 days, after which colonies >50 cells were counted. The results confirmed a similar cytotoxicity of Cu(phen)(NO_3_)_2_ for both GBM cell lines and suggested this cytotoxicity to be higher than that exerted by the HSAFt–Cu(phen) adduct (Fig. 6b). This further corroborates observations made with healthy and with colorectal cancer cell lines: HSAFt–Cu(phen) is cytotoxic, but the protein cage seems to exert a shielding effect on the cells from its cargo.

The effect of Cu(phen) presence within HSAFt on the cytotoxicity of TMZ was assessed next. The HSAFt–Cu(phen)–TMZ formulation showed improved activity compared to TMZ against both glioma cell lines, overcoming the MGMT resistance mechanism expressed by U373M with a GI_50_ ∼ 13 μM (Fig. 5). However, this increased activity was also observed in normal fibroblasts, a consequence of the presence of Cu(phen) in the formulation. Clonogenic assays performed on U373M showed that HSAFt–Cu(phen)–TMZ caused a dramatic decrease in cell survival compared to TMZ-only treatment (Fig. 6c). Furthermore, almost no colonies were stained after 13 days of incubation for both sensitive (U373V) and resistant (U373M) GBM cells.

## Experimental

### Materials

All the commercially available reagents were purchased from Sigma-Aldrich (Merck). Buffers and aqueous solutions were prepared using Milli-Q grade water. Commercially available reagents for chemical syntheses were used throughout, without purification unless otherwise stated. In cell culture experiments, media components were sterilised by filtration (0.2 μm filter). Protein concentrations were determined by Bradford assay on a Fluostar OPTIMA plate reader (BMG Labtech) using BSA as standard. Protein mass spectra were acquired using a Bruker Ultraflex III MALDI-TOF analyser. UV-vis spectra were acquired using quartz cuvettes on a UV-2600 with a CPS-100 cell positioner (Shimadzu) and with Agilent Cary 8454 UV-vis spectrometer. The multi-elemental analysis was undertaken using ICP-MS iCAP-Q (Thermo Fisher Scientific). Absorbances for cell viability assays were measured using a PerkinElmer plate reader. High-resolution transmission electron microscopy (HRTEM) images were acquired using a JEOL 2100F TEM microscope. The copper-1,10-phenanthroline complex Cu(phen)(NO_3_)_2_ was prepared as previously reported.^22^ GraphPad Prism (v. 10.1.2) was used to run statistical analysis tests, which were typically unpaired *t*-tests. Values are reported as mean ± SD and significant differences are considered when *p* < 0.05.

### Preparation of AFt from horse spleen ferritin (HSAFt)

The AFt protein cage used for this work was prepared from 3 batches of HSFt purchased from Sigma-Aldrich as saline solutions.

A sample of the stock HSFt protein (∼50 mg mL^−1^) was diluted 10 times into sodium acetate buffer (0.1 M NaOAc, pH 5.5) and transferred into a dialysis bag (MWCO 14000 Da). The protein solution was then immersed in acetate buffer (0.1 M NaOAc, pH 5.5, 1.5 L) and purged with nitrogen for 15 min before the addition of 0.03 M of thioglycolic acid (2 mL) into the dialysis buffer. The protein was dialysed for 2 h under constant purge with N_2_ and then 1 mL of thioglycolic acid (0.03 M) was added into the dialysis buffer. After one hour the protein was transferred into a fresh sodium acetate buffer (0.1 M NaOAc, pH 5.5, 1.5 L) and the procedure (thioglycolic acid addition and subsequent dialysis) was repeated 6 times, keeping the solution at room temperature. After that, the protein was finally transferred into 1.5 L of fresh sodium acetate buffer and dialysed for another 24 h to remove any residual thioglycolic acid. The protein was then split into 1 mL aliquots and stored at −20 °C. The protein concentration was determined by Bradford assay.^40^

### Encapsulation of copper-1,10-phenanthroline within HSAFt

The Cu(phen)(NO_3_)_2_ complex was loaded into the HSAFt cage by the nanoreactor route, *via* diffusion through the protein channels. The metal complex and the protein cage (24 mer) were mixed in different ratios up to a maximum of 22 000 to 1 ratio (Cu to 24 mer) in 0.1 M sodium acetate buffer (pH 5.5) or 50 mM glycine buffer (pH 7.4) for a total incubation time of 3 h. The HSAFt solution (3 mL, 3.5 μM) was stirred at room temperature while 0.5 mL of the Cu(phen)(NO_3_)_2_ solution (stock solution 57.2 mM) was added every 30 min to avoid HSAFt precipitation. After incubation, the free Cu(phen)(NO_3_)_2_ complex was removed by overnight dialysis against >500 volumes of 0.1 M NaOAc pH 5.5 or 50 mM glycine pH 7.4 buffer, which was exchanged twice to ensure copper removal. The 50 mM glycine pH 7.4 buffer was supplied with 10 mg of CHELEX resin to ensure the efficiency of excess Cu(phen) removal. The samples were stored at −20 °C for further analysis. Copper content was assessed by ICP-MS analysis and UV-vis spectrometry. The integrity of the protein cage was confirmed by native PAGE, DLS and HRTEM. Samples were prepared at protein cage concentration of 0.3–0.5 mg mL^−1^ (0.68–1.1 μM) for UV-vis, 0.05 mg mL^−1^ (0.1 μM) for DLS and 1 μM for HRTEM, using freshly prepared encapsulation buffer (0.1 M NaOAc pH 5.5 or 20 mM glycine pH 7.4).

### Encapsulation of temozolomide within the HSAFt–Cu(phen) structures

The anticancer alkylating agent TMZ was loaded into the HSAFt–Cu(phen) cages by the nanoreactor route, *via* diffusion through the protein channels. The encapsulation procedure was performed using HSAFt–Cu(phen) samples prepared at pH 5.5 and stored for 2 weeks at −20 °C to allow enough time for the formation of the copper adducts. The drug and the protein cage (24 mer) were mixed in 800 to 1 ratio in 0.1 M sodium acetate buffer (pH 5.5) for a total incubation time of 3 h. The TMZ solution (stock 10 mM, 7.2 μmol added) was prepared in DMSO and was added to the HSAFt solution every 30 min, with constant stirring at 4 °C to prevent precipitation of the test agent. The solution was then filtered through an Amicon ultra-4 centrifugal filter (MWCO 10000 Da) at 4000 × *g* for 4 min. The final TMZ concentration was estimated by UV-vis spectroscopic analysis, at the absorbance maximum of 330 nm and using the extinction coefficient of 9800 M^−1^ cm^−1^.^22^ HSAFt concentration was confirmed by Bradford assay.^40^ The integrity of the cage was confirmed by native PAGE, DLS and HRTEM. Samples were stored at −20 °C for further analyses.

### Cargo release studies from HSAFt–Cu(phen)

For drug release studies, 250 μL of HSAFt nanoformulation (stock 1 μM) were added into a D-Tube Dialyzer Mini 12–14 kDa MWCO (Novagen) and samples were dialysed at 37 °C and mixed at 150 rpm against 1 mL of either pH 5.5 NaOAc buffer (0.1 M) or pH 7.4 phosphate-buffered saline (PBS). After 1, 3, 5, 7, and 24 h of dialysis, drug leakage in the dialysis solution was monitored by UV-vis spectroscopy at 268 nm. The estimation of percentage release was determined by assuming as absolute maximum the absorbance value of Cu(phen) in the initial HSAFt–Cu(phen) sample. Release experiments were performed in triplicate.

### High-resolution transmission electron microscopy studies

The unstained samples for HRTEM analysis were diluted to an HSAFt concentration of 1 μM in sodium acetate buffer, deposited on a graphene oxide grid on holey carbon film, left to dry for 10 minutes, washed with one droplet of deionised water and dried under vacuum overnight before analysis. An aliquot from the same sample was applied on a separate carbon grid, stained with 2% uranyl acetate and dried at room temperature overnight before imaging.

### 3-(4,5-Dimethylthiazol-2-yl)-2,5-diphenyltetrazolium bromide (MTT) assay

HCT-116 human-derived colorectal carcinoma (CRC) cells, MRC5 foetal fibroblasts, U373V (MGMT−) and U373M (MGMT+) GBM cells were seeded at a density of 5 × 10^3^ cells per well into 96-well microtiter plates and allowed to adhere for 24 h before treatment with test compounds HSAFt, Cu(phen)(NO_3_)_2_, TMZ, HSAFt–Cu(phen), HSAFt–Cu(phen)–TMZ, solvent and buffer controls. The seeding density was 400 cells per well for HCT-116, MRC5 and 650 cells per well for GBM cell lines when tested with the anticancer agent TMZ. Serial dilutions were prepared in medium supplemented with 10% foetal bovine serum (FBS), before each assay. Viable cells at the time of drug addition (time-zero; *T*_0_), following 72 h drug exposure and 6 days drug exposure (for TMZ and HSAFt–Cu(phen)–TMZ only) were determined by adding MTT (2 mg mL^−1^) in PBS solution to reach a final concentration of 400 μg mL^−1^. Incubation of the plates for an additional 2–3 h at 37 °C allowed reduction of MTT by viable cell dehydrogenases to insoluble formazan product. Supernatants were aspirated from the wells and formazan solubilised with 150 μL DMSO. Absorbance was read at 570 nm using a plate reader (PerkinElmer). Absorbances were then corrected by subtraction of the solvent background and used to calculate the estimated concentration causing reduction of cell viability by 50% (GI_50_) for both cell lines. Control cell growth was determined using the control groups at *T*_0_ after 24 h incubation as references. The final GI_50_ values were estimated through the curve-fit analysis of GraphPad Prism, using the non-linear regression function.

### Cell count assay

The cell lines tested (MRC5 and HCT-116) were seeded at a density of 1 × 10^4^ cells per well in 0.5 mL medium in 12-well plates and incubated at 37 °C for 24 h. On day 2 each well was treated with 0.5 mL of the test agents diluted in medium. The treatments used for this assay were HSAFt, Cu(phen)(NO_3_)_2_, HSAFt–Cu(phen) at the final concentration of 0.5 μM. After 72 h of exposure to the treatment the well content was aspirated, and the cells were resuspended with 0.25 mL of trypsin. Trypsin was inactivated by adding 0.25 mL of medium and a single cell suspension was produced by putting the content of each well through a syringe needle. The cells were then counted using a haemocytometer.

### Clonogenic assay

Clonogenic assays were set up in 6-well plates and U373V, U373M were seeded at 400 cells per well. The cells were incubated at 37 °C for 24 h before treatment with AFt (0.05 μM), Cu(phen)(NO_3_)_2_ (2.5 μM), HSAFt–Cu(phen) (2.5 μM Cu), TMZ (20 μM) or HSAFt–Cu–TMZ (20 μM TMZ). The cells were exposed to the test compounds for 6 days. The media were then removed, the cells were washed with ice cold PBS and fresh medium was added into the wells. Plates were incubated at 37 °C until colonies of ≥50 cells were observed in control wells. Culture media were removed, colonies were washed in ice cold PBS, fixed with 100% methanol, stained with 0.05% methylene blue and counted. Duplicate repeats for each test agent were performed in at least three independent trials.

## Conclusions

This study investigated in detail the effects of loading HSAFt with the metal complex Cu(phen)(NO_3_)_2_, the precursor of a family of novel anticancer agents currently under clinical evaluation. TEM analysis of the protein metal complexes showed the formation of copper adducts visible for up to 4 months. The investigation of the composition of these adducts highlighted the presence of organised structures possibly formed by the interaction between copper and amino acid residues inside the protein coexisting with a central bulk composed of copper phenanthroline complexes. DLS and zeta potential results showed that the HSAFt cage remained intact, with no indication of charged compounds associated with its external surface. When tested under physiological conditions (37 °C, PBS pH 7.4), the HSAFt–Cu(phen) samples showed loss of up to 40% of the phenanthroline ligand within 10 h of incubation and of 10% of the copper in 24 h.

*In vitro* cell growth inhibitory and cytotoxicity assays showed that both the free complex Cu(phen)(NO_3_)_2_ and the Cu(phen) encapsulated within HSAFt were cytotoxic to colorectal cancer cells HCT-116, as well as to glioblastoma multiforme cell lines U373V and U373M. However, they also showed cytotoxicity against the non-tumourigenic cell line MRC5. No dramatic change in the cell toxicity measured by GI_50_ values was observed by encapsulating the complex within HSAFt. On the other hand, colony formation assays suggested that the protein cage could exert a shielding effect on the cells from its cytotoxic cargo. From HSAFt–Cu(phen) it was possible to produce a formulation containing both Cu(phen) and the prodrug TMZ encapsulated within AFt. This formulation demonstrated greater activity against GBM cells compared to TMZ alone, however the presence of the metal also contributed to more potent activity toward healthy MRC5 fibroblasts, a result that requires further investigation to assess the clinical viability of this system. Further *in vivo* studies are also planned to interrogate drug delivery capabilities in more clinically relevant settings.

## Author contributions

MLC conducted the research, collected the data, performed the analysis and wrote the original draft. MF and LT supported the HRTEM analysis of apoferritin samples. TB, NRT and AP formulated the research project and supervised the student. LT, TB, NRT and AP reviewed and edited the final draft.

## Conflicts of interest

There are no conflicts to declare.

## Supplementary Material

RA-014-D3RA07430G-s001
