# Supplementary material for: Encapsulation of copper phenanthroline within horse spleen apoferritin: characterisation, cytotoxic activity and ability to retain temozolomide
Source: RSC Adv. 2024 Apr 29;14(20):14008–16. doi: 10.1039/d3ra07430g (PMC11056943; doi:10.1039/d3ra07430g)
Supplement: RA-014-D3RA07430G-s001 [file RA-014-D3RA07430G-s001.pdf]

Electronic Supplementary Material (ESI) for RSC Advances.
This journal is © The Royal Society of Chemistry 2024

Supplementary Information

Encapsulation of copper phenanthroline within horse spleen apoferritin: characterisation, cytotoxic activity and ability to retain temozolomide

Maria Letizia Cassioli,[a] Michael Fay,[b] Lyudmila Turyanska,[a] Tracey D. Bradshaw,[c] Neil R. Thomas[d] and Anca Pordea*[a]

a Faculty of Engineering, University of Nottingham, NG7 2RD, United Kingdom.

b Nanoscale and Microscale Research Centre, University of Nottingham, NG7 2RD, UK.

c Biodiscovery Institute, School of Pharmacy, University of Nottingham, NG7 2RD, UK.

d Biodiscovery Institute, School of Chemistry, University of Nottingham, NG7 2RD, UK

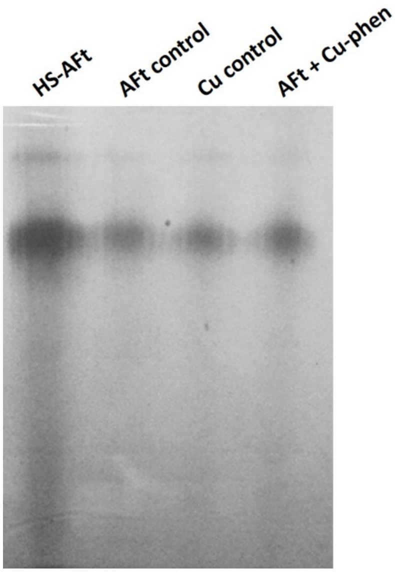

Figure SI-1. Native PAGE of HSAFt-Cu(phen) after encapsulation at pH 5.5. Lane 1: HSAFt reference; Lane 2: HSAFt-Cu(phen).

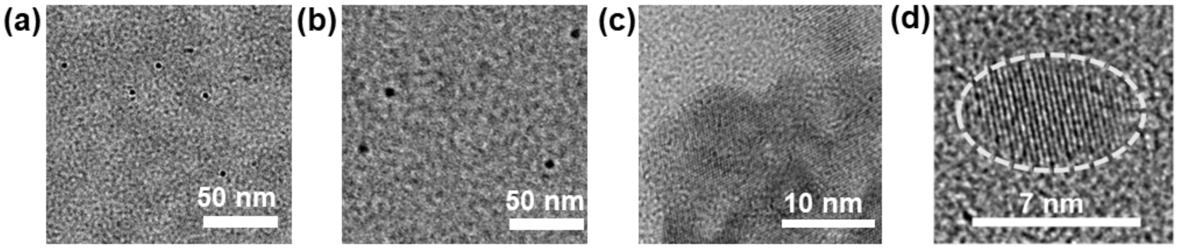

Figure SI-2. HRTEM image of HSAFt-Cu(phen) samples showing the growth of the copper cores at (a) 1 week, (b) 4 months, (c) 6 months and (d) the crystalline organisation. Samples were prepared at 1 μM after encapsulation at pH 5.5.

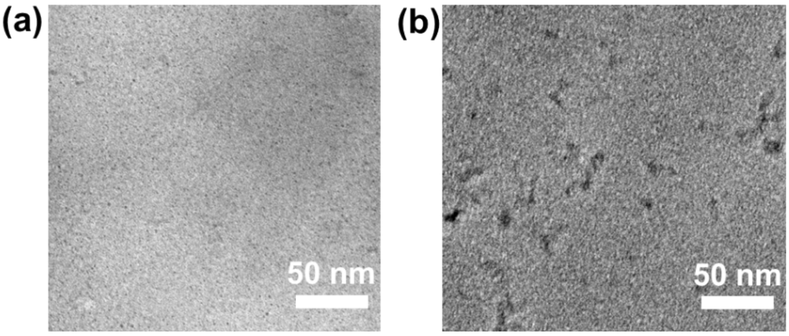

Figure SI-3. HRTEM images of (a) HSAFt-Cu and (b) HSAFt-phen in NaOAc buffer pH 5.5 at 1 μM, taken one week after the encapsulation.

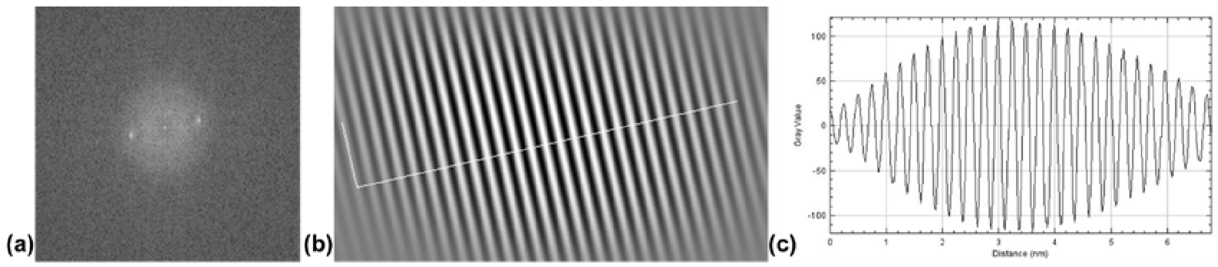

Figure SI-4. Lattice fringes spacing estimation of the HSAFt-Cu(phen) adducts. The measurement of the lattice fringes was performed using the image analysis tool ImageJ. The original image (Figure SI-2d) was processed by Fourier transformation (a) from which a representation of the lattice fringes based on their intensity from the original image (b) was obtained. By drawing a section perpendicular to the fringes on image b it is possible to produce a plot with the intensity profile of the fringes (c). The number of peaks shown on the plot corresponds to the number of fringes present in the perpendicular segment drawn. The ratio between the line length (nm) and the number of peaks gives the nm estimate of the atoms spacing which can be used to determine the unit cell arrangement.

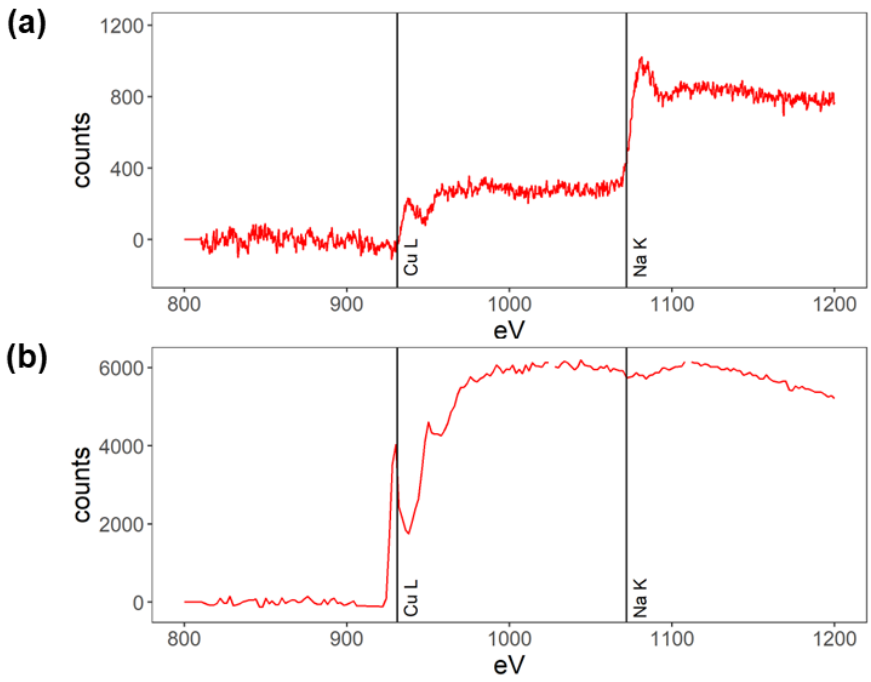

Figure SI-5. EELS spectrum of (a) AFt-Cu(phen) compared to (b) a reference spectrum of CuO thin film. The spectrum of AFt-Cu(phen) sample (a) shows a very low-intensity signal in the 900-950 eV region which corresponds to the energy edge produced by copper oxide as it can be identified in the reference spectrum (b), supporting evidence of copper present as CuO.

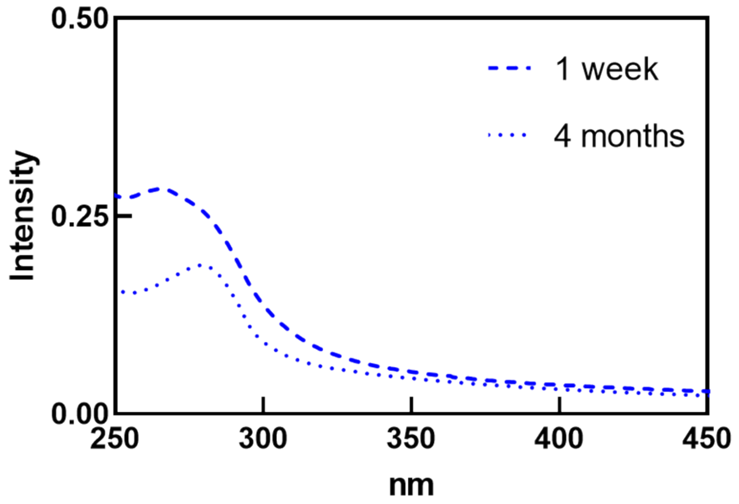

Figure SI-6. UV-visible spectrum of HSAFt-Cu(phen) sample produced at pH 7.4 (1 µM).

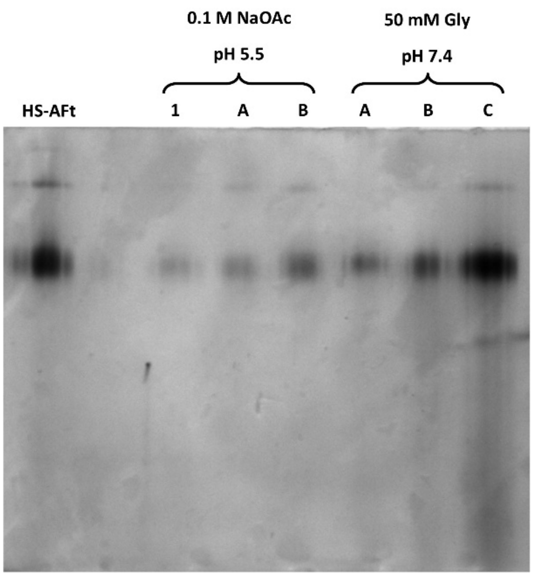

Figure SI-7. Native PAGE of HSAFt-Cu(phen) sample produced at pH 7.4. Lane 1: HSAFt, Lane 2: HSAFt-Cu(phen).

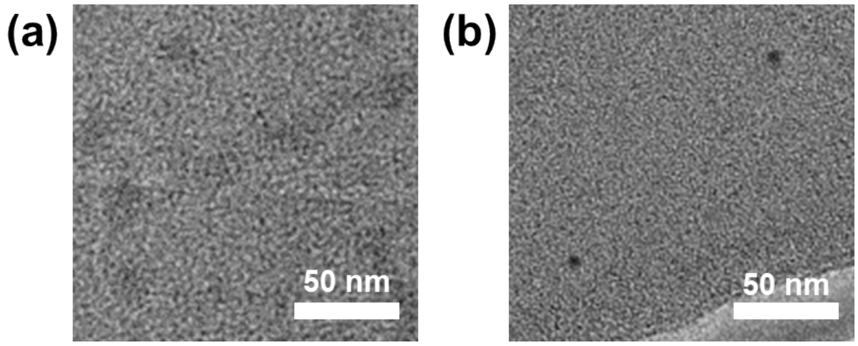

Figure SI-8. HRTEM of HSAFt-Cu(phen) sample produced at pH 7.4. (a) HSAFt-Cu(phen) sample imaged after 1 week from the encapsulation, where no structures can be observed; (b) HSAFt-Cu(phen) sample imaged after 4 months from the encapsulation, showing some structures, which could not be confirmed as crystalline copper organisations.

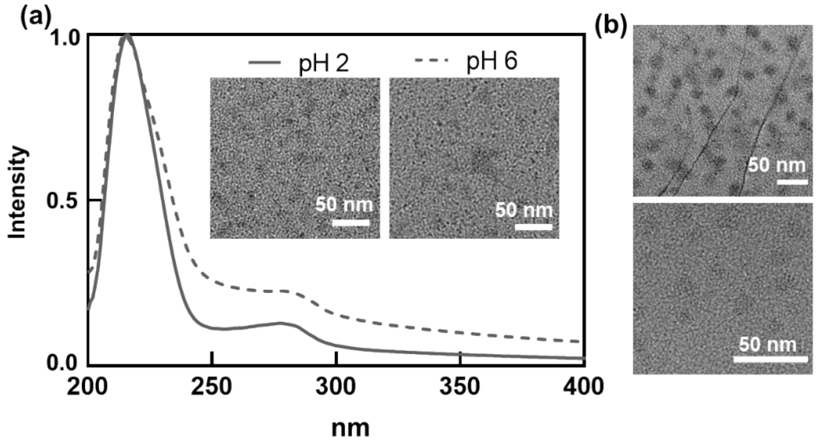

Figure SI-9. (a) UV-visible spectra and HRTEM of HSAFt-Cu(phen) prepared at pH 7.4 and then exposed to pH 2 and pH 6 to test for Cu adducts formation. (b) HRTEM images of HSAFt-Cu(phen) encapsulated at pH 7.4 and exposed to 37 °C (top) and 50 °C (bottom) to test for Cu adducts formation.

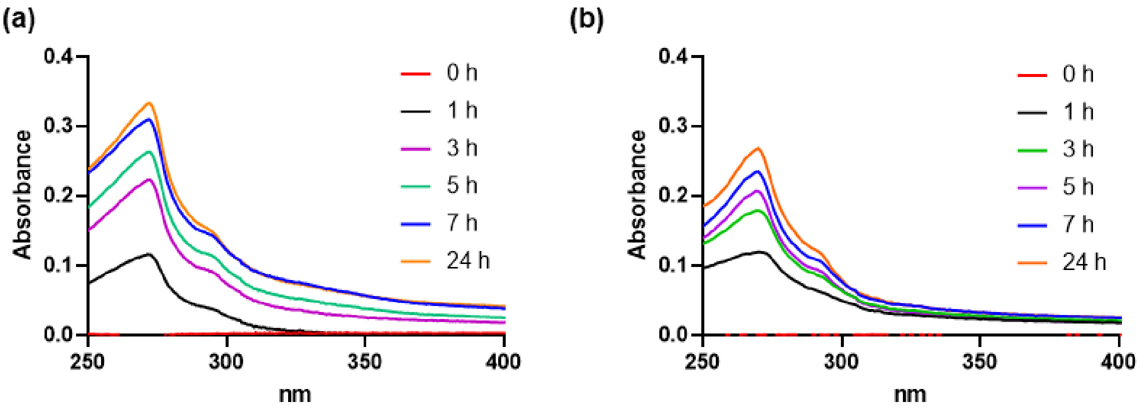

Figure SI-10. UV-visible spectra of the HSAFt-Cu(phen) dialysis solutions of the release test at (a) pH 5.5; (b) pH 7.4.

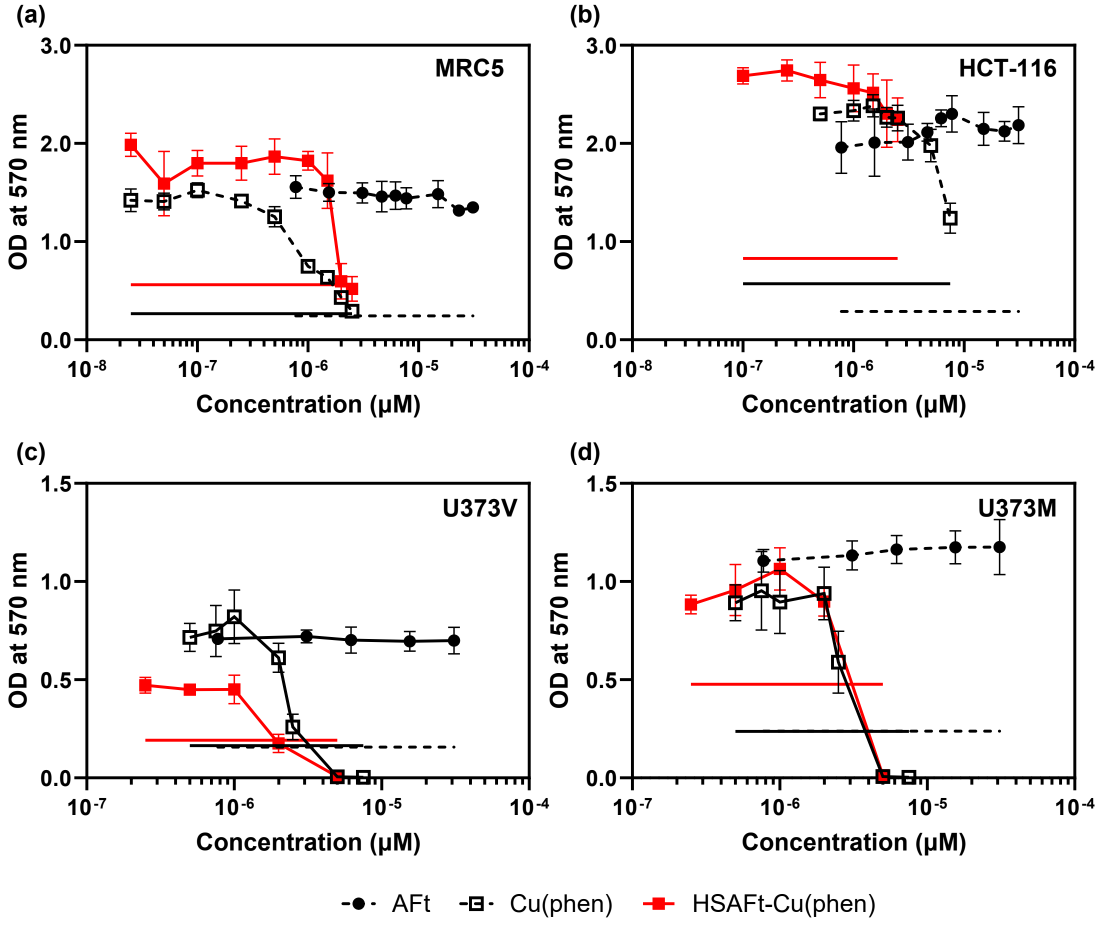

Figure SI-11. Effect of HSAFt, $Cu(phen)(NO_3)_2$ and HSAFt-Cu(phen) on healthy and cancer cell lines.

The outcome of the MTT assay was determined by the number of viable (metabolising) cells after exposure to the test compound. The MTT reagent was metabolised to formazan by

mitochondrial dehydrogenases of viable cells. Blue formazan crystals were then solubilised and absorbance at ~550 nm measured, which was directly proportional to the number of viable cells. For HSAFt-Cu(phen), concentrations are estimated based on the copper content in the sample. MTT assays were performed at time of test agent addition (T_0) and following 72 h treatment of cells with test agents. Representative dose-response curves are shown. Data points are mean ± SD (n=8); 3 independent trials were conducted**.** The data collected were normalised using the absorbance values recorded 24 hrs after seeding the cells, before treatment (T_0). The normalised results were then plotted as the percentage of viable cells over the different treatment dilutions.

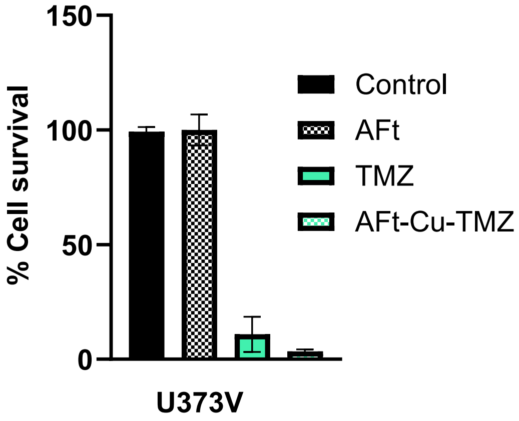

Figure SI-12. Clonogenic assays with GBM cell lines. Cells were seeded in 6-well plates at a low density (400 cells/well) and incubated with test agents (HSAFt (0.05 µM), Cu(phen) (2.5 µM), HSAFt-Cu(phen) (2.5 µM Cu), TMZ (20 µM) or HSAFt-Cu(phen)-TMZ (20 µM)) until day 6, when the cells were then washed and incubated in fresh media until colonies were observed in the control wells.

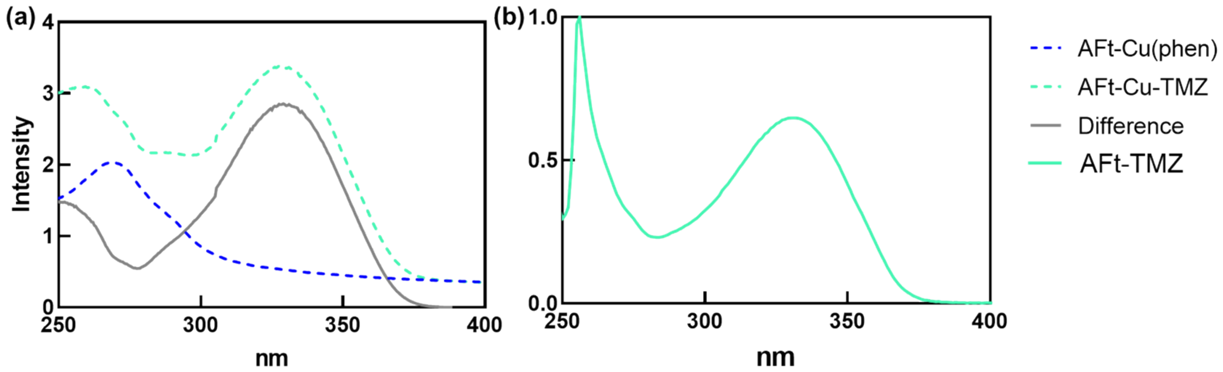

Figure SI-13. UV-vis spectra of (a) HSAFt-Cu(phen) and HSAFt-Cu(phen)-TMZ. The difference was used for estimation of TMZ concentration; (b) HSAFt-TMZ at 1 µM. λmax(TMZ) = 330 nm; ε_{330}(TMZ) = 9800 M^{-1} cm^{-1}.

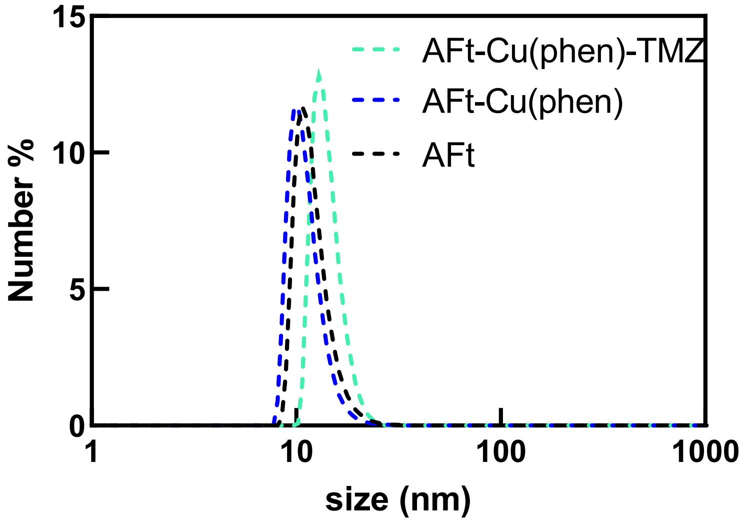

Figure SI-14. Hydrodynamic size distribution of AFt, AFt-Cu(phen) and AFt-Cu(phen)-TMZ measured by dynamic light scattering (left) and their corresponding zeta potential values (right).

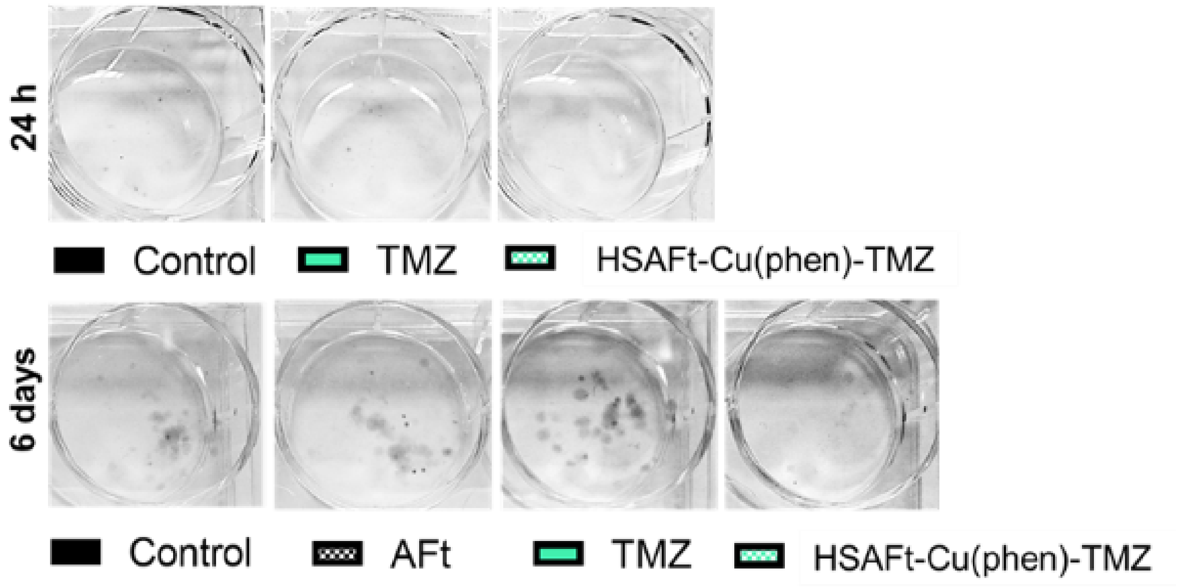

Figure SI-15. *In vitro* activity effect of AFt-Cu(phen)-TMZ, TMZ and AFt on glioblastoma MGMT+ (U373M), after 24 h and 6-days exposure.

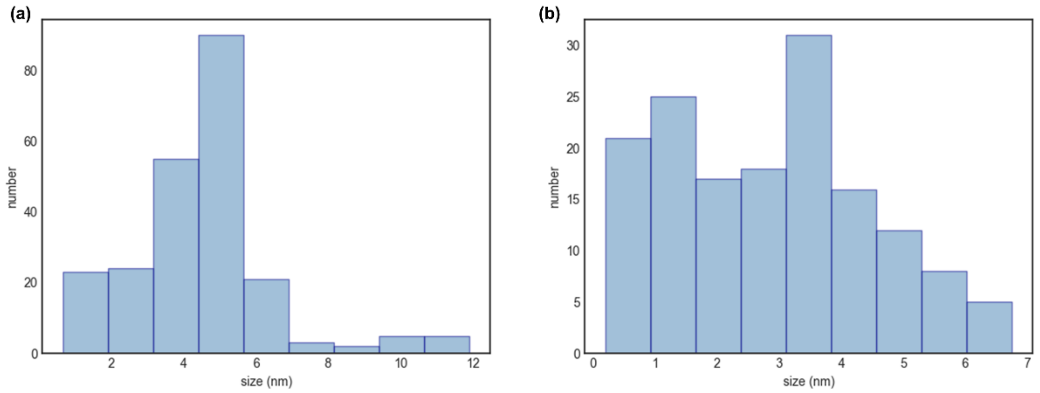

Figure SI-16. Histograms from the TEM images of the (a) HSAFt-Cu(phen) and (b) HSAFt-Cu(phen)-TMZ samples. Average particle sizes are 8.4 +/- 3.4 nm for (a) and 2.9 +/- 1.6 nm for (b).
